# Microstructure and Mechanical Properties of Dissimilar Friction Stir Welded Joint AA7020/AA5083 with Different Joining Parameters

**DOI:** 10.3390/ma15051910

**Published:** 2022-03-04

**Authors:** Janusz Torzewski, Magdalena Łazińska, Krzysztof Grzelak, Ireneusz Szachogłuchowicz, Janusz Mierzyński

**Affiliations:** 1 Faculty of Mechanical Engineering, Military University of Technology, Gen. S. Kaliskiego 2 Str., 00-908 Warsaw, Poland; krzysztof.grzelak@wat.edu.pl (K.G.); ireneusz.szachogluchowicz@wat.edu.pl (I.S.); janusz.mierzynski@wat.edu.pl (J.M.); 2 Faculty of Advanced Technologies and Chemistry, Military University of Technology, Gen. S. Kaliskiego 2 Str., 00-908 Warsaw, Poland; magdalena.lazinska@wat.edu.pl

**Keywords:** friction stir welding, dissimilar alloys joining, microstructure, mechanical properties

## Abstract

The present paper aims to analyze the influence of process parameters (tool traverse speed and tool rotational speed) on the macrostructure, microhardness, and mechanical properties of dissimilar friction stir welded (FSW) butt joints. Nine combinations of FSW parameters welded joints of aluminum alloys 7020-T651 and 5083-H111 were characterized. Plates in 5 mm thickness were welded using the FSW method as dissimilar joints with three values of tool rotation parameters (400, 800, and 1200 rpm) and three welding speeds (100, 200, 300 mm/min). The macroscopic observations revealed various shapes of the stir zone and defects resulting from excess and insufficient heat input. Microfractographic analysis and tensile test results showed that the samples made with the FSW parameters of 800 rpm and 200 mm/min had the best strength properties: UTS = 303 MPa, YS = 157 MPa, and A = 11.6 %. Moreover, for all welds at welding speed 100 mm/min, the joint efficiency reached 95%.

## 1. Introduction

Aluminum has an exciting combination of properties (i.e., low density, adequate strength, easy to manufacture) that can be developed and modified by alloying and processing. Mainly, aluminum alloys are classified as non-heat-treatable and heat-treatable alloys. The first group includes pure aluminum alloys (series 1xxx) and alloys with the primary alloying element: manganese (3xxx series), silicon (4xxx series), and magnesium (5xxx series). The second group consists of aluminum alloys with the main additives: copper (2xxx series), magnesium and silicon (6xxx series), and zinc (7xxx series) [1,2]. The observed increase of interest in aluminum alloys in many industries like aerospace, shipbuilding and automotive also applies to the possibility of combining different alloys in one structure. Joining dissimilar materials is challenging but essential for many applications as the advantages of different materials can be well exerted. Friction stir welding (FSW) enables joining dissimilar materials, including not heat-treated 5xxx with hard-to-weld 7xxx aluminum alloys [3,4,5,6,7]. For FSW, two parameters are critical: tool rotation speed (ω, rpm) and tool traverse speed (v, mm/min) along the joint line [8,9,10]. Additionally, there are considerations concerning the analysis of the influence of the tool pin geometry and offsetting of the tool on the strength properties of dissimilar joints [11,12,13]. The rotation of the FSW tool results in stirring and mixing of joined materials around the rotating pin. The transfer of the tool proceeds the stirred material from the front to the back of the pin and determines the speed of the welding process.

This research examined the effect of two main factors, tool rotational speed and tool transverse speed, on the macrostructure and mechanical properties of dissimilar friction stir welds. The examination characterizes the welds between high mechanical strength (7020-T651) materials and high corrosion resistance (5083-H111) of commercial aluminum alloys. AA7020 presents poor weldability by traditional fusion joining methods due to the presence of copper; on the other hand, AA5083 is amenable to joining with many standard electric and resistance welding techniques. Consequently, the application of FSW seems to be an adequate technique to obtain good quality welds for this material combination. Among the recently published articles on friction stir welding, there are publications containing research on the 5xxx and 7xxx Al series. Dong et al. [9] conducted microstructural tests of dissimilar AA7003/AA6060 aluminum alloys joints prepared using FSW. The welding process was accomplished at a rotational speed of 1000 r/min and a welding speed of 40 mm/min. The authors concluded that the weak area exists in the heat-affected zone (HAZ) of 6060 alloys, which was placed on the retreating side during FSW and the joint’s ultimate tensile strength (UTS) reached 159.2 MPa. Ahmed et al. [14] considered the influence of the tool traverse speed on microstructure, hardness, and strength properties of dissimilar FSW joints between 5083-H111 and 7075-T6 alloys. Still, the authors did not observe an explicit effect of increasing the welding speed on grain size, hardness, or tensile strength. The ultimate tensile strengths of welds varied between 245 and 267 MPa. Another work by Ahmed et al. [10] analyzed the metallographic aspects, and the hardening behavior of dissimilar joints friction stir welded AA5083-O and AA5754-H14. It is worth noting that the authors obtained the best results for the tool rotation speed (400 rpm) and tool traverse speed (60 mm/min) among the three studied combinations of manufacturing parameters. The ultimate tensile strength reached 224 MPa, which is about 96% of the strength of the weaker material in the joint. The microstructure and mechanical properties of friction stir welded dissimilar butt joints of 6061/7050 aluminum alloys were examined by Rodriguez et al. [15]. The aluminum alloys were joined at three different tool rotational speeds (270, 340, and 410 rpm) and the welding transverse speed was set at 114 mm/min. In this study, experimental results indicated a noticeable growth in the yield and the ultimate tensile strength of the joint as the tool rotational speed was raised. The most satisfactory joint strength was obtained for the highest rotational speed (410 rpm), including an average ultimate tensile strength (UTS) of 192.6 ± 1.5 MPa. Shojaeefard et al. [16] focused on FSW joints’ microstructure and mechanical properties in 7075-O and 5083-O alloys. The object of the research was also the optimization of the friction stir welding process using a neural network and a particle swarm algorithm. The FSW joint, made at the tool rotation speed of 1400 rpm and welding speed of 20 mm/min, was characterized by the highest strength (267 MPa). The influence of the FSW process parameters and the tool profile on the mechanical properties of AA 5082 and AA 6061 welds was investigated by Raman G. et al. [17]. Authors obtained weld joints of similar and dissimilar materials at tool rpm of 1600 and 2600 with a feed of 15 and 20 mm/min. For dissimilar AA 5082 and AA 6061 joints, weldments have exhibited improved mechanical properties at lower rpm, i.e., 1600 rpm and higher feed rates of 20 mm/min (UTS equal to 157 MPa).

According to the previously published papers, it can be concluded that researchers can obtain correct dissimilar FSW joints of alloys in the 7xxx and 5xxx series. Research is still being carried out on the influence of the welding process parameters on the properties of these joints, in terms of the possibility of shortening the joining process by increasing the welding speed without deteriorating the properties strength of the joint. As well as analyzing the combined effect of the FSW parameters using the heat input ratio. Therefore, this study aims to better understand the correct selection of process parameters (i.e., tool rotation speed, tool traverse speed) that guarantees solid connections between alloys 7020 and 5083 with appropriate mechanical properties.

## 2. Material and Experimental Procedures

In this study, 5083-H111 and 7020-T651 aluminum alloys were used as base materials. The research was carried out on plates with 5 mm thickness, obtained according to the rolling direction with the following dimensions: Length × Width × Height = 500 mm × 100 mm × 5 mm. The AA 5083-H111 is a non-heat treatable alloy, and plastic deformation is the main hardening mechanism in this alloy. The H111 condition was obtained with some work hardening by shaping processes, but less than that required for an H11 temper. The 7020 aluminum alloy is a heat treatable alloy that age hardens naturally. The T651 heat treatments contain: supersaturation—heating up to 430 °C for 45 min, cooling with water of min 15 °C, natural ageing for 0–6 days at 20 °C and artificial ageing—120 °C/96 h.

The analyzed aluminum alloys’ chemical composition and basic mechanical properties are presented in Table 1 and Table 2, respectively.

All friction stir welds were performed at the Military University of Technology using ESAB FSW Legio 4UT machine (ESAB, Warsaw, Poland). All sets were performed with the tool position control with a constant tilt angle set to 2° and axial force of approximately 17 kN. MX Triflute tool geometry consisted of a threaded conical pin (diameters from 6.5 to 8.7 mm and height 4.8 mm) and a spiral shoulder with a diameter of 19 mm for nine different sets of welding parameters (Table 3).

**Figure 1:**
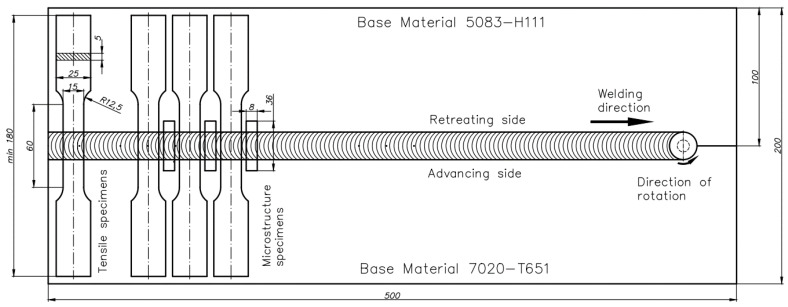
Arrangement of different aluminum sheets at the time of making FSW joints and dimensions of specimens used for tensile and microstructure investigations.

The connections were made at three different welding speeds (100, 200, and 300 mm/min) and the rotational speeds of the tool (400, 800, and 1200 rpm). Different aluminum alloys were joined in a butt configuration with AA7020 on the advancing side (AS) and AA5083 on the retreating side (RS). The dimensions of the sheets and the method of taking samples from the joined metals are shown in Figure 1.

The FSW direction was parallel to the rolling direction of the sheet. The sheets of base materials were rigidly attached to the worktable of the Legio 4UT (ESAB, Gothenburg, Sweden) machine. For the microstructure study, samples were cut perpendicular to the welding direction (cross-welds) and then mechanically grinding on SiC papers and polished with 3- and 0.25-μm diamond suspensions. The process of preparing the specimens was completed by chemical polishing with the use of 0.1 µm colloidal silica suspension. For metallographic observations of the microstructures by light-optical microscopy, the samples were etched with Keller’s reagent (2 mL HF (48%), 3 mL HCl (conc.), 5 mL 63% HNO_3_, 190 mL H_2_O) for 1 min, and Graff-Sargent reagent (3 g CrO_3_ + 87.5 ml H_2_O + 15 mL 63% HNO_3_ + 1 mL 40% HF) for 2 min. A qualitative analysis of the macro- and microstructure obtained samples was performed using a Nikon MA 2000 optical microscope (Nikon, Leuven, Belgium) equipped with an image analyzer NIS-Elements BR 5.30.01. The Vickers microhardness distribution measurements were on the cross-section of polished samples with 100 g load and 10 s loading time in every indentation. Vickers indentations along three parallel lines: the top line (1 mm from the top of the weld), the middle line (at 2.5 mm from the top of the weld), and the bottom line (at 4 mm) were used to carry out microhardness distribution analysis (the distance of indentations in each line was 0.15 mm). Tensile tests were carried out at room temperature and a constant displacement rate of 4 mm × min^−1^ using servo-hydraulic testing machine INSTRON type 8802. Tensile tests were performed on samples (Figure 1) made following ISO 6892-1: 2019, and strain measurements were made using an extensometer with a gauge length of 50 mm.

## 3. Results

### 3.1. Macro and Microstructural Investigation

Table 4 presents surface morphologies and FSW joints macrostructures of AA7020/5083 depending on welding parameters produced with the Triflute pin. Observations of the upper surface of welds for most of the joints were characterized by barely noticeable semicircular features and clear ribbon flashes. The lack of bonding on the top surface of the joints was observed only in the case of the highest tool rotational speed (1200 rpm) and the welding speed (300 mm/min).

FSW joints macrostructures were made for eight connection cases, as an external defect was observed for the A7A5-3-12 connection, and we did not analyze this connection further. Macroscopic observation reveals differences in the macrostructures depending on welding parameters and reveals inner defects in certain welds.

The welds 7020–5083, produced at the welding speed of 100 mm/min and the rotational speeds of 400, 800, and 1200 rpm, were characterized by a different degree of mixing. Sample A7A5-1-4 shows the lowest degree of mixing; moreover, microscopic observations reveal a slight tunnel defect at the bottom of the joint. The tunneling defect arose in the sample with the lowest tool rotational speed. Lower heat input effects in higher flow stresses, resulting in insufficient material movement deterring material consolidation. The remaining welds in this configuration show good mixing of the alloys in the welding center. The joint at 800 rpm allows it to be most accessible to distinguish the characteristic shape of the FSW joints. The FSW 7020/5083 joints produced at the rotational speeds of 400 and 800 rpm and constant welding sped of 200 mm/min were characterized by an appropriate degree of mixing. On the other hand, the observation of the FSW joint photo taken at the same welding speed and the rotational speed of 1200 rpm (A7A5-2-12) reveals an inner defect in the central part of the joint. The cavity defect was not be seen on the surface. A further increase in the feed rate (300 mm/min) resulted in defective FSW 7020–5083 connections for all speeds. Void defects created at this level of the feed speed result from insufficient heat input, and it can be concluded from this that the tool traverse speed is too high, concerning the adopted rotational speed. The highest welding speed used in the research led to wormhole defects due to insufficient material flow and lack of joint filling. Due to the previously rejected sample A7A5-3-12, only two joint pictures are included in Table 4.

**Figure 2:**
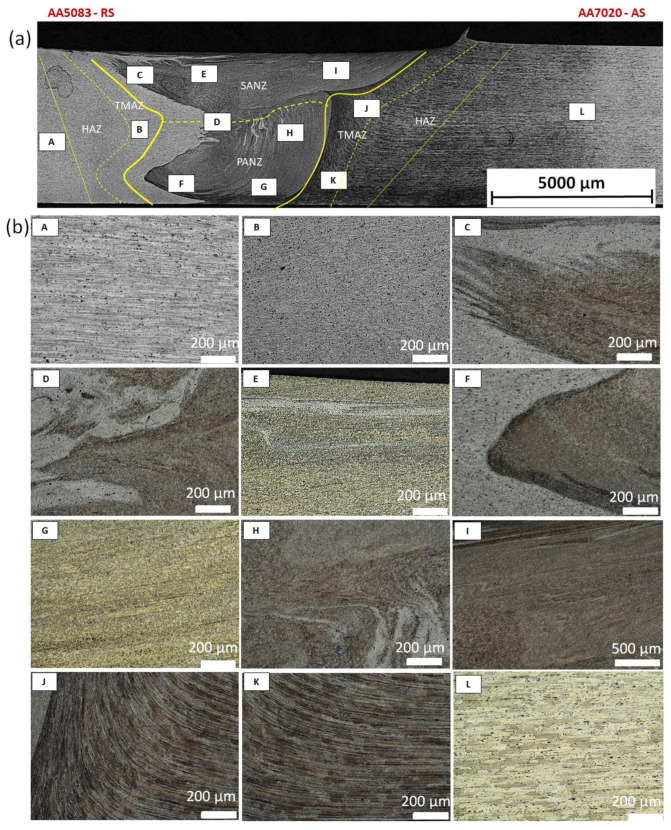
Microstructure of boundary between TMAZ, SANZ, and PANZ in A7A5-2-8 sample (**a**) macrograph of the cross-section with the joint zones marked, (**b**) zooms of some characteristic areas of the weld: A—base material from the side AA5083, B—TMAZ/HAZ from the side AA5083, C—SANZ from the side AA5083, D—SANZ/PANZ border from the side AA5083, E—SANZ center, F—PANZ from the side AA5083, G—PANZ from the side AA7020, H—SANZ/PANZ border from the side AA7020, I—SANZ from the side AA5083, J,K—TAMZ/HAS from the side AA7020.

An example of the macrostructure of the sample A7A5-2-8 FSW joint of AA5083 and AA7020 with characteristic zones is shown in Figure 2. This joint was prepared under a tool rotational speed of 800 rpm and a transverse welding speed of 200 mm/min. The macro-observations carried out showed that both sides of the weld are not symmetrical due to the nature of the process. The microscopic observations showed that there was no real mixing of the materials was achieved. Only a small amount of material from one side is moved onto the other side of the nugget [18]. Various zones can be observed in the weld, which is the effect of the temperature gradient across the weld cross-section and the large uniform deformation [19].

**Figure 3:**
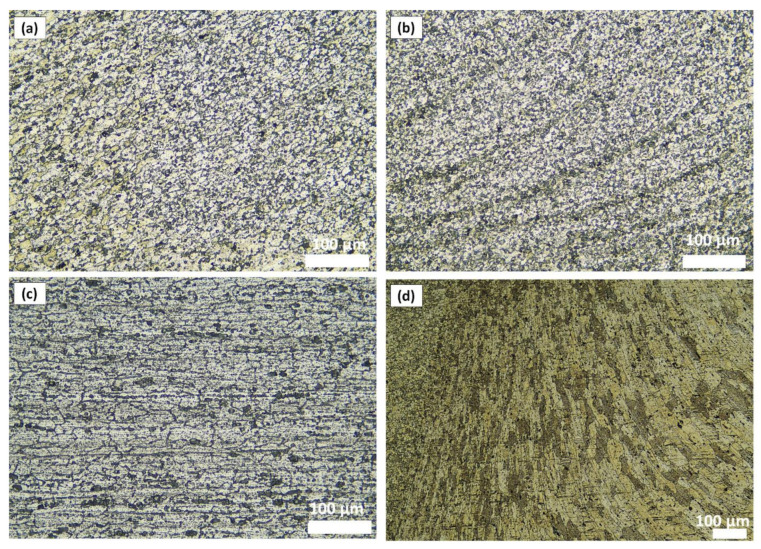
Microstructure of (**a**) SANZ, (**b**) PANZ, (**c**) TMAZ + HAZ from the side AA7020, (**d**) TMAZ + HAZ from the side AA5083 in A7A5-2-8 sample.

On the basis of microscopic observations, the occurrence of different regions: heat-affected zone (HAZ), the thermo-mechanical affected zone (TMAZ), the shoulder-affected nugget zone (SANZ), and the pin-affected nugget zone (PANZ) on both sides of the weld was found. The photos from the optical microscope (Figure 2) show that the boundaries between the different zones of the weld and the base materials are clearly identified through the whole thickness. The SANZ zone corresponds to about the top third of the nugget. On the other hand, the PANZ zone corresponds to about two-thirds of the lower nugget. A similar shape of the weld was obtained by Bertrand et al. [15] for the AA2XXX/AA7XXX joint. Determining the boundary between TMAZ and HAZ based on optical microstructural observation is difficult. It is almost impossible to locate the end of the HAZ towards the base material. The boundary between the TMAZ zone and the PANZ/SANZ zone is easy to define due to the equal size and morphology of the grain (Figure 2—details C, F, J). The significant grain refinement can be presented by comparison of base materials and PANZ and SANZ microstructure (Figure 3).

The base material microstructure has grain size about 40–60 µm and 30–50 µm for AA7020 (Figure 3c) alloy and AA5083 alloy (Figure 3d), respectively. Ultrafine grain is formed in the nugget zone (Figure 3a,b). This is a characteristic of this joining technique due to the dynamic recrystallization phenomenon. The grain size in the PANZ and SANZ zones is 5–10 µm. Deformed, elongated grains present in TMAZ (Figure 3d) visualize a material flow around the tool during the welding process.

### 3.2. Microhardness Behavior

**Figure 4:**
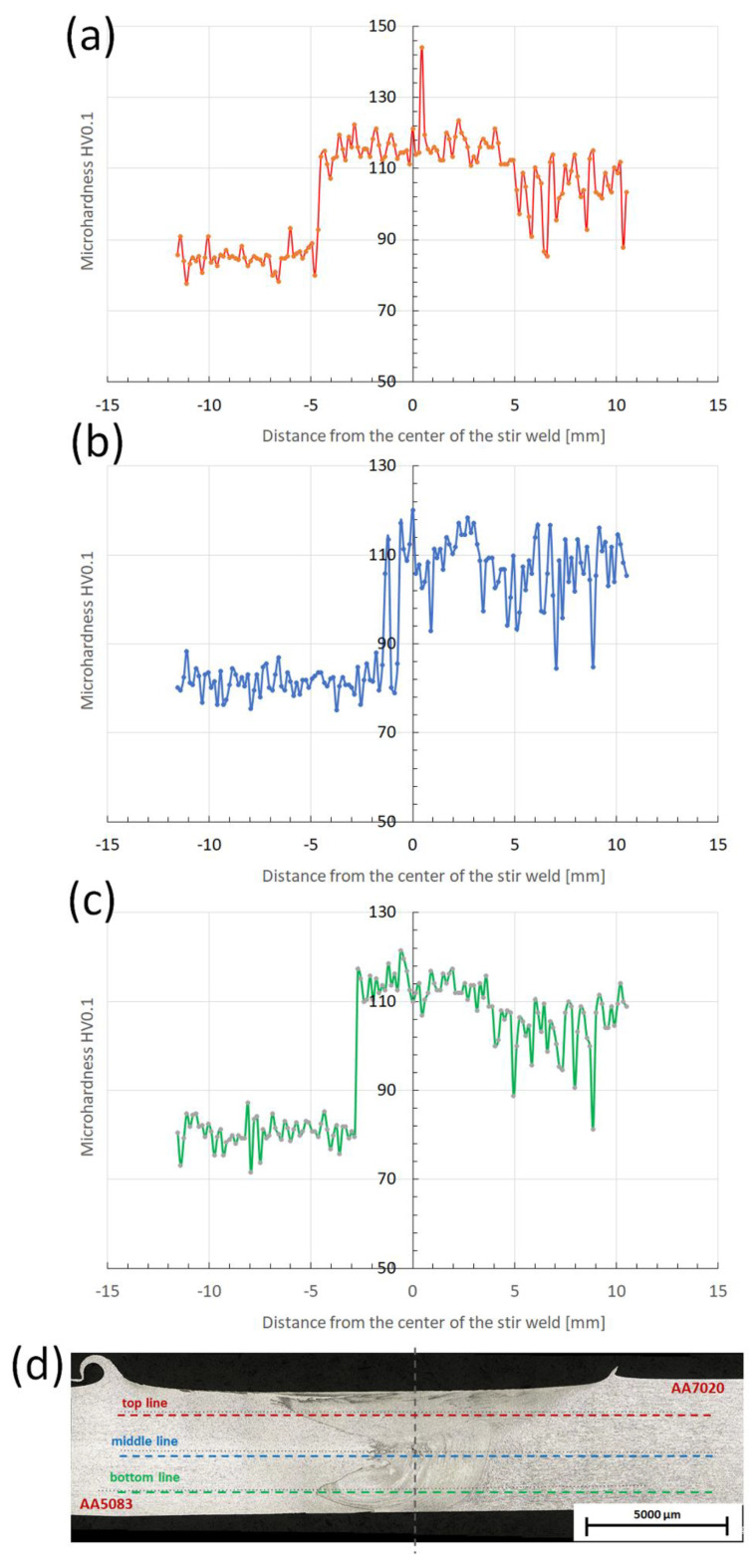
Microhardness profile across the stir weld in A7A5-2-8 sample (**a**) top line of measurement, (**b**) middle line of measurement, (**c**) bottom line of measurement, (**d**) measurement scheme.

The results of Vickers’ microhardness tests on the cross-section of connections for three different measurement lines: top, middle and bottom are shown in Figure 4.

The microhardness measurements depending on the position of the measuring line differed little for the base material and the heat-affected zone. The base material hardness is 86 HV0.1 and 103 HV0.1 for AA5083-H111 and AA7020-T651, respectively. It was observed that there was no decrease in the hardness of the base materials in all heat affected-zones for both alloys and for three profiles. However, a slight increase in the hardness of AA7020 alloy can be observed towards the center of the weld regardless of its position in AS or RS. The mean microhardness value for AA7020 alloy in the nugget zone (SANZ and PANZ region) was 120 HV0.1. Regardless of the position of the measuring line, a hardening process occurred in the SANZ and PANZ region, and the hardness value of the base material was a bit lower. A similar effect was noticed by the authors of the paper [3]. Ahmed et al. demonstrated that Vickers micro-hardness for AA7020 alloy in the nugget zone is 120–130 HV1. This effect can be explained by the solid solution strengthening that can occur when stirring the dissimilar alloys [11]. Moreover, in the area of the welding nucleus, the formation of equiaxial fine grains was observed (Figure 3), which is the result of intensive plastic deformation at elevated temperature and dynamic recrystallization [4]. The fine-grained microstructure improves the mechanical properties of stir weld [20]. Although the increase in hardness in FSWed aluminum alloys is rare, also Mishra and Ma [4] referred to studies showing a slight increase in hardness across the weld nucleus compared to TMAZ for slightly (less than 6%) hardened 5083-H112 as a result of a very fine grain size created by FSW. On the other hand, for the AA5083 alloy, a slight decrease in hardness towards the center of the weld from the AS position side can be observed, which results from the thermal softening cycle of the FSWed alloy [3].

### 3.3. Tensile Properties

Table 5 summarizes the ultimate tensile strength (UTS), 0.2% yield strength (YS), and elongation at break (A) test results of the dissimilar FSW joints. Additionally, the weld efficiency was calculated as the ratio of the tensile strength of the weld to the tensile strength of the weaker aluminum alloy, in this case, AA5083-H111.

The analysis of the tensile test results of dissimilar FSW joints (Table 5) shows that excellent results were obtained for four welding parameters. For all joints at a tool traverse speed of 100 mm/min along with one joint at a welding speed of 200 mm/min and for 800 rpm rotation speed, the weld efficiency was 95% and above.

**Figure 5:**
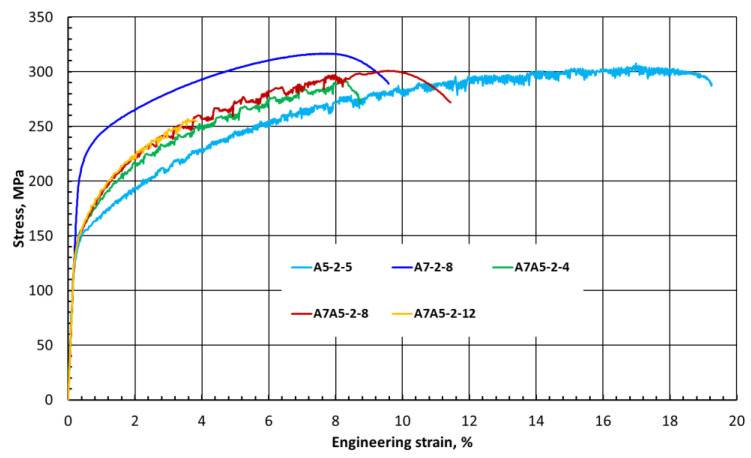
Representative stress–strain curves for selected welds at the same welding speed (200 mm/min).

The maximum tensile strength and yield strength (303 and 157 MPa, respectively) are achievable for the A7A5 2-8 sample produced at the best welding parameters. Notably, the elongation of defect-free welds was above 8.6%, which is the maximum elongation for AA7020-T651 (Table 2). Moreover, the elongation of the A7A5 2-8 sample reached the value of 11.6%, which exceeds one of the base alloys by 3%. The FSW welds made at the welding speed of 300 mm/min were characterized by a much lower strength than the joints for the other welding parameters. For sample A7A5-3-8, the welding efficiency was only 40%, which is consistent with previous observations revealing imperfections in the microstructure of the joints (Table 4), which contributed to the deterioration of the mechanical properties. Figure 5 shows representative stress–strain curves of similar (A5-2-5, A7-2-8) and dissimilar (A7A5-2-4, A7A5-2-8, A7A5-2-12) welds of base materials AA7020 and AA5083 made at a welding speed of 200 mm/min. The test results marked A5-2-5 and A7-2-8 relate to the tensile tests of the previously made own research [21]. Comparing the stress–strain curves of similar and dissimilar welded joints at the same welding speed (200 mm/min) shows that properly selected joining parameters allow the joints to obtain a tensile strength on the level of weaker material in dissimilar joints. In addition, the engineering strain of the dissimilar joint (A7A5-2-8) was obtained, exceeding the properties of the similar weld of AA7020 (A7-2-8).

## 4. Discussion

For friction stir welding technology, two fundamental parameters are tool rotation speed, ω (rpm) and tool traverse speed, v (mm/min) along the joint line. The rotation of the tool results in stirring and mixing of material around the rotating pin, and the translation of the tool moves the stirred material from the front to the back of the pin and finishes the welding process [4,22,23]. The FSW method generates a large amount of heat due to friction between tools and workpieces and an intense pressure leading to plastic deformation at the contact of the rotating tool. These factors cause the temperature to rise significantly in and around the stirred zone. A simultaneous approach to these two factors has been proposed by Seung-Ju et al. [24], assuming constant contact pressure. The heat input during FSW can be expressed as the following equation (Equation (1)):

HIR = ω/v(1)

where HIR is the heat input ratio, ω (rpm) is the tool rotation speed, and v (mm/min) is tool traverse speed.

Another proposal to consider the two essential parameters of the welding process was formulated as the heat input index [25,26], which is a candidate for representing the average thermal profile during welding. The Heat Input Index (HI) was described by equation (Equation (2)):

HI = (ω^2^/v) × 10^−4^, (2)

where HI is the heat input index, ω (rpm) is the tool rotation speed and v (mm/min) is tool traverse speed. The discussed ratios were calculated for each trial and presented in Table 6.

The sample with the best parameters and the pieces with the same Heat Input Ratio (HIR) are marked green in Table 6. These studies show that HIR does not always correctly represent the effect of welding parameters on strength properties. Comparing the test results of three samples with the same heat input ratio of four (Table 6) reveals a different effect. The selected samples include A7A5-2-8 with the best strength properties, samples A7A5-3-12, which was rejected from the strength tests due to a defect, and sample A7A5-1-4 with medium strength properties. Similarly, Kasman et al. [27] conducted analyses of the constant ω/v ratio for connections of dissimilar alloys and obtained both correct and defective welds. The samples with the same HIR index have a different Heat Input Index (HI), which is 0.32 for the sample with the best parameters, with average values of 0.16, the piece disqualified from the tests 0.48. Likewise, in this case, it is difficult to find a simple relationship between the HI index value and the quality of the joint. Hence, it follows that using the compared coefficients without their practical verification or taking into account additional factors may result in obtaining poor connections, especially in the case of joining different materials.

### 4.1. Effect of Tool Rotational Speed

**Figure 6:**
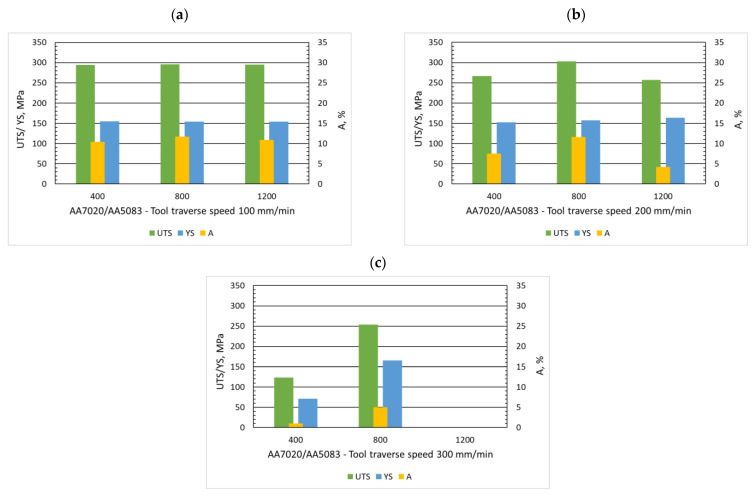
Influence of rotational speed (400, 800, 1200 rpm) on ultimate tensile strength (UTS), yield strength (YS), and elongation (A) with three traverse speeds (100 mm/min, (**a**), 200 mm/min (**b**) and 300 mm/min (**c**)).

One of the principal parameters of the FSW process is the tool rotational speed, which influences the process of mixing materials and the occurrence of defects. These studies show that the rotational speed of the tool determines the obtaining of a good quality joint, but it cannot be considered regardless of the welding speed. In Figure 6, it can be observed that for a constant tool traverse speed of 100 mm/min, the change of the rotational speed from 400 to 1200 rpm practically does not cause changes in the strength properties. Similar observations were made by Shojaeefard et al. [16] for the rotation speed variation from 700 to 1200 rpm. On the other hand, Kasman et al. [28] reported results showing a reduction in ultimate tensile strength with increasing rotational speed from 1000 to 1250 rpm.

The situation is different for a constant tool traverse speed of 200 mm/min, where the change in rotational speed affects the strength properties of the dissimilar joints tested. At a rotational speed of 800 rpm, the highest tensile strength (303 MPa) and elongation (11.6%) were obtained, and when the rate increased to 1200 rpm, the joint strength decreased by almost 50 MPa and elongation by 7.4%. No, K. et al. [29] observed a similar variation in strength properties when changing the rotational speed at a fixed level of the welding speed dissimilar aluminum alloys for both 180 and 240 mm/min.

Motivated by the increase in productivity and the successful FSW joint tests presented in the literature [29,30,31], the tests were carried out at a welding speed of 300 mm/min with various rotational speeds (400, 800, 1200 rpm). At the this welding speed, defects in the form of tunnel voids and wormholes appeared regardless of the rotational speed. The wormhole defect resulted in the rejection of samples from further tests for the rotational speed of 1200 rpm. For the remaining rotational speeds (400, 800 rpm), tunnel defects significantly reduced the strength properties by even more than 50% (Figure 6) in relation to the best dissimilar joint (A7A5-2-8).

### 4.2. Efect of Tool Traverse Speed

**Figure 7:**
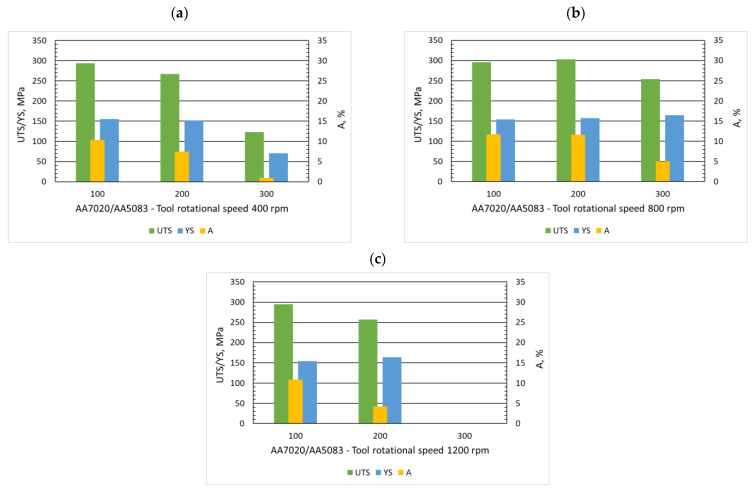
Influence of tool traverse speed (100, 200, 300 mm/min) on ultimate tensile strength (UTS), yield strength (YS), and elongation (A) with three rotational speed (400 rpm (**a**), 800 rpm (**b**) and 1200 rpm (**c**)).

The second equally important parameter of the FSW process is the tool traverse speed, which affects the distribution process of mixed materials and determines the time of friction heat exposure. Our research shows that the welding speed affects the strength properties of FSW joints, but this parameter should be considered together with the rotational speed of the tool. In Figure 7, it can be observed that for a constant rotational speed of 400 rpm, an increase in the welding speed from 100 mm/min to 300 mm/min significantly reduces the strength properties. The tensile strength decreased from 294 to 123 MPa, and the yield strength dropped more than twice, as did the elongation at the breakpoint. Ramachandran et al. [32] noticed a decrease in tensile strength and elongation with just a 10 mm/min increase in welding speed for a tool rotation speed of 500 rpm.

For a constant rotational speed of 800 rpm, changes in strength properties due to an increase in the tool traverse speed are not as straightforward as the previous speed. Similar analyses concerning the influence of the tool traverse speed on the strength properties for this tool’s rotational speed and different welding rates can be found in the literature [33,34,35]. The highest strength properties in this group were achieved by samples (A7A5-2-8) produced at a welding speed of 200 mm/min, for which the tensile strength value reached 303 MPa and elongation 11.6%. For the feed speed of 100 mm/min, the strength properties are slightly lower (below 4%), while for 300 mm/min, the tensile strength decreased by almost 20% and the elongation by over 50%. At the highest rotational speed (1200 rpm), only for samples produced at a welding speed of 100 mm/min, no defects were observed, reflected in the best strength properties (UTS—295 MPa, YS—154 MPa, and elongation 10.9%). At subsequent feed rates, defects were observed that weakened the samples or were qualified as defective in the case of a speed of 300 mm/min. The article by Guo et al. [36] shows the results of excellent connections of 7075-6061 alloys at welding speeds of 120, 180, and 300 mm/min.

## 5. Conclusions

In the present study, dissimilar FSW joints of alloys in the 7020-T651 and 5083-H111 was produced. Using FSW samples with butt joint configuration, the influence of changing process parameters (tool rotation speed and tool traverse speed) on microstructure and mechanical properties of FSW joints was evaluated. This paper delivered further information on the correct selection of the FSW process parameters (i.e., tool rotation speed and feed rate) that provides sound joints between 7020 and 5083 alloys. The investigation also reports new observations on the heat input coefficients taken into account when selecting parameters for FSW joining.

The following conclusions can be figured out based on the research carried out and the discussion of the results.

1. Incorrect selection of the welding speed and rotational speed of the tool leads to the formation of tunnel defects and wormhole defects, which were revealed despite the correct looking joint surfaces. The lack of bonding on the top surface of the joints was observed only in the case of the highest tool rotational speed (1200 rpm) and the highest welding speed (300 mm/min).
2. The microscopic observations showed that complete mixing of the materials in the joint was not achieved for the tested alloys. Nevertheless, the strength properties were satisfactory and comparable to the weaker material in the joint: UTS = 303 MPa, YS = 157 MPa, and A = 11.6%.
3. It was observed that there was no decrease in the hardness of the base materials in all heat affected-zones for both alloys and three profiles. Regardless of the position of the measuring line, a hardening process occurred in the welding region, and the Vickers micro-hardness for AA7020 alloy in the nugget zone reached values above 110 HV0.1.
4. The best obtained 7020/5083 FSW joints showed up to 98% joint efficiency with the viewpoint of ultimate tensile strength weaker material in the joint (5083-H111) and exhibited almost 12% elongation, which exceeds the elongation of the base material (7020-T651).
5. These studies show that the main parameters of the FSW process (tool rotational speed and tool traverse speed) cannot be considered separately, and their mutual influence on each other is difficult to describe with a simple relationship, especially concerning dissimilar joints. Motivated by the increase in productivity, the increase in the feed speed while maintaining a constant heat input ratio did not ensure obtaining the correct FSW joint.

The presented results of the paper constitute the basis for further analyzes related to the influence of other parameters, such as, e.g., tool offset on the quality of joining materials with different thermal conductivity.

## Figures and Tables

**Table 1:** Chemical composition of the aluminum alloys used in the current study (wt. %).

Alloy	Al	Mg	Mn	Si	Fe	Cr	Zn	Ti	Cu
AA5083	Bal.	**4.34**	0.63	0.076	0.13	0.064	0.035	0.055	0.032
AA7020	Bal.	1.30	0.24	0.16	0.32	0.14	**4.7**	0.034	0.05

**Table 2:** Mechanical properties of basic materials according own research.

Alloy	Tensile Strength (MPa)	0.2% Yield Strength (MPa)	Elongation (%)	Microhardness Hv0.1
AA5083-H111	310	165	20.2	82
AA7020-T651	390	300	8.6	107

**Table 3:** Summary of welding matrix.

Processing Parameter		Tool Rotational Speed, ω (rpm)		
		400	800	1200
Tool traverse speed, v (mm/min)	100	A7A5-1-4	A7A5-1-8	A7A5-1-12
	200	A7A5-2-4	A7A5-2-8	A7A5-2-12
	300	A7A5-3-4	A7A5-3-8	A7A5-3-12

**Table 4:** Surface morphologies and FSW joints macrostructures of AA7020/AA5083 depending on welding parameters.

Sample Designation	Surface Morphology	FSW Joints Macrostructure
A7A5–1–4	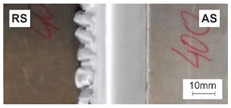	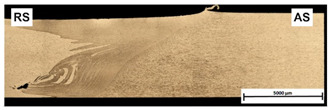
A7A5–1–8	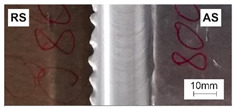	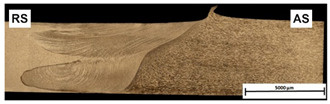
A7A5–1–12	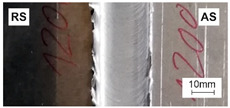	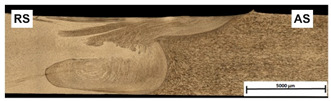
A7A5–2–4	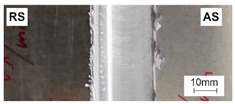	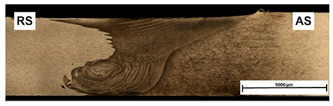
A7A5–2–8	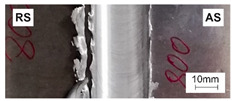	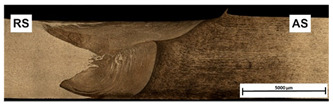
A7A5–2–12	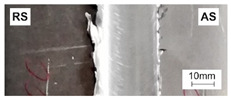	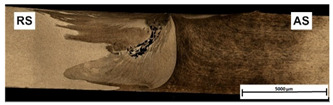
A7A5–3–4	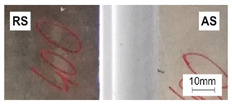	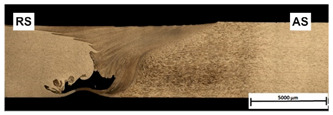
A7A5–3–8	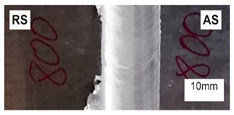	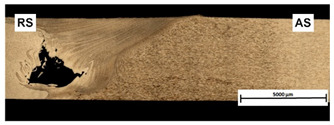

**Table 5:** Comparison of mechanical properties and weld efficiency of FSW AA7020/AA5083 welds (standard deviations of the results in brackets).

Sample Designation	Tensile Strength (MPa)	0.2% Yield Strength (MPa)	Elongation (%)	Weld Efficiency (%)
A7A5-1-4	294 (±3.2)	155 (±1)	10.4 (±1.7)	95
A7A5-1-8	296 (±6.1)	154 (±3.5)	11.7 (±0.8)	95
A7A5-1-12	295 (±0.1)	154 (±0.6)	10.9 (±0.2)	95
A7A5-2-4	267 (±25)	152 (±1.7)	7.5 (±4.5)	86
A7A5-2-8	303 (±1.8)	157 (±1.1)	11.6 (±0.2)	98
A7A5-2-12	256.6 (±7.8)	164 (±0.5)	4.2 (±0.3)	83
A7A5-3-4	123 (±24)	71 (±26.2)	0.95 (±0.05)	40
A7A5-3-8	254 (±47.7)	165 (±0.7)	5 (±3.6)	82

**Table 6:** Heat input ratio for each sample with the designation.

Sample Designation v-ω	A5A7-1-4	A5A7-1-8	A5A7-1-12	A5A7-2-4	A5A7-2-8	A5A7-2-12	A5A7-3-4	A5A7-3-8	A5A7-3-12
Heat Input Ratio	4	8	12	2	4	6	1.3	2.6	4
Heat Input Index	0.16	0.64	1.44	0.08	0.32	0.72	0.053	0.213	0.48

## Data Availability

The data presented in this study are available on request from the corresponding author.
